# The Neural Correlates of Desire

**DOI:** 10.1371/journal.pone.0003027

**Published:** 2008-08-27

**Authors:** Hideaki Kawabata, Semir Zeki

**Affiliations:** 1 Wellcome Laboratory of Neurobiology, Department of Cell and Developmental Biology, University College London, London, United Kindgom; 2 Department of Psychology, Kagoshima University, Korimoto, Kagoshima, Japan; University of Granada, Spain

## Abstract

In an event-related fMRI study, we scanned eighteen normal human subjects while they viewed three categories of pictures (events, objects and persons) which they classified according to desirability (desirable, indifferent or undesirable). Each category produced activity in a distinct part of the visual brain, thus reflecting its functional specialization. We used conjunction analysis to learn whether there is a brain area which is always active when a desirable picture is viewed, regardless of the category to which it belongs. The conjunction analysis of the contrast *desirable > undesirable* revealed activity in the superior orbito-frontal cortex. This activity bore a positive linear relationship to the declared level of desirability. The conjunction analysis of *desirable > indifferent* revealed activity in the mid-cingulate cortex and in the anterior cingulate cortex. In the former, activity was greater for desirable and undesirable stimuli than for stimuli classed as indifferent. Other conjunction analyses produced no significant effects. These results show that categorizing any stimulus according to its desirability activates three different brain areas: the superior orbito-frontal, the mid-cingulate, and the anterior cingulate cortices.

## Introduction

Desire is defined in the *Oxford English Dictionary* as “that feeling or emotion which is directed to the attainment of some object from which pleasure or satisfaction is expected”. The definition is well suited as an introduction to this study, for it has three elements that are critical to it, namely an external object, a feeling directed to the attainment of satisfaction from that object, and an internal (subjective) reward or satisfaction. We therefore hypothesized that desire would activate at least some segment of the reward system of the brain, especially areas that are involved with both expectation and satisfaction. Previous studies have attempted to clarify the neural correlates of emotional states related to reward and motivation, such as monetary reward [1]–[3], drug craving [4], [5], and food choice [6], [7], without however identifying a common brain area whose activity correlates with these different categories of reward. In the present study, we investigated desire in general, without confining ourselves to a particular category, for example desire for persons. Our aim was to learn whether there are any brain areas in which activity correlates with desire in general and, if so, whether the level of activity in them is related quantitatively to the declared levels of desire.

## Methods

### Subjects

Eighteen healthy, right-handed, Japanese subjects (nine females) in the 20 to 48 yr age range participated in the study. All had normal or corrected-to-normal vision, and none had a history of neurological or psychiatric disorder. Informed written consent was obtained from all subjects. The study was approved by the Ethics Committee of the National Hospital for Neurology and Neurosurgery, London, UK, and the Ethics Committee of the Institute of Advanced Technology Research, Japan.

### Stimuli and prior scaling test

**Figure 1:**
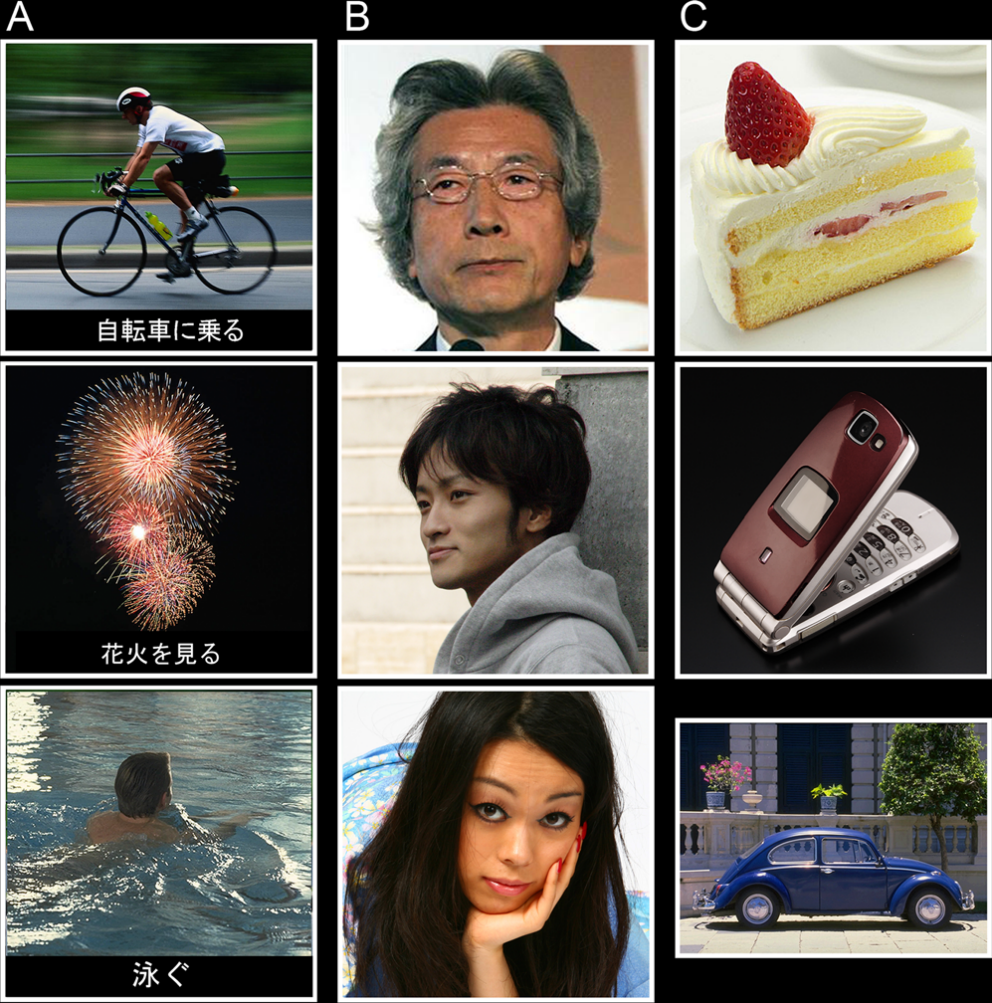
Examples of stimuli for (A) ‘events’ (upper, cycling; middle, fireworks; lower, swimming) (B) ‘persons’ (upper, politician; middle, anonymous male; lower, anonymous female), and (C) ‘objects’ (upper, cake; middle, mobile phone; lower, car). Only events stimuli had subtitles.

We wanted to ensure that the scanned subjects would classify roughly equal proportions of the viewed stimuli as being ‘desirable’, ‘indifferent’ or ‘undesirable’. To do so, we obtained normative data from fifty subjects who did not take part in the scanning (25 male and 25 female Japanese psychology or education students aged between 20 and 22). Although these subjects were not especially matched to those taking part in the scanning experiment in terms of age or sociological metrics, behavioral data (see below) collected from the scanning group nevertheless shows roughly equal proportions of ‘desirable’, ‘indifferent’ and ‘undesirable’ responses when averaged across all subjects. The scaling subjects viewed 432 pictures of three stimulus categories (144 pictures each of events, persons and objects). The ‘events’ category included pictures of, for example, ‘a traffic jam’, ‘a trip to south island’, ‘playing football’, with explanations in words given as subtitles. The ‘persons’ category consisted of pictures of famous people (e.g., actors, politicians, etc) and also of anonymous ones, divided between males and females. The ‘objects’ category included pictures of cars, foods and adornments (e.g. jewellery, fashion accessories etc.) (see Fig. 1). Subjects were shown each picture for 3s with an interval of 2s between pictures and each picture was given a desirability score between 1 and 10. Scores of 1–4 were classified as ‘undesirable’, 5 and 6 as ‘indifferent’, and 7 and above as ‘desirable’. We then selected the stimuli to be used for the scanning experiment. For each of the three categories (events, persons and objects) we selected 24 ‘desirable’, 24 ‘indifferent’ and 24 ‘undesirable’ pictures (where the desirability classification was the common decision of at least 50% of the subjects). This resulted in a total of 216 images viewed by each scanning subject.

### fMRI stimulus and procedure

The stimuli were back-projected onto a screen viewed through an angled mirror. The resolution of the screen was 1,024×768 pixels, and the height of each stimulus was about 18° (600 pixels) while its width varied. We used an event related design, in which pictures were presented in random order, and therefore unpredictably. Each stimulus was classified as ‘desirable’, ‘indifferent’, or ‘undesirable’ by pressing one of three buttons. Each of the 216 stimuli was presented for 3s (no fixation required). After each stimulus presentation, subjects fixated a central cross on a blank screen for 1s. All instructions were in Japanese, and subjects were asked to push a button with the right hand to classify each stimulus as ‘hoshii (or nozomashii)’, ‘dochirademonai’, or ‘hoshikunai (or nozomashikunai)’, Japanese words corresponding to ‘desirable’, ‘indifferent’, or ‘undesirable’. We emphasize at the outset that we are studying a complex sentiment, which has at least two components, ‘wanting’ and ‘preference’. The two, though usually linked, are not always necessarily so. In our instructions, we therefore strongly emphasized to subjects that their classification should be based on desirability, and not on preference, giving them the example of their preferred food, which they may not desire when on a full stomach. Subjects were interviewed to confirm that they understood the distinction before proceeding with the experiment (see also Discussion).

### fMRI data acquisition and analysis

Each subject was scanned in a 1.5-T MRI scanner (Shimazu-Marconi, Japan) to obtain blood oxygen level-dependent (BOLD) contrast functional images, using a gradient-echo EPI sequence (TE, 50 ms; TR, 4 s). Each functional image volume of the whole brain was acquired in 40 slices, each consisting of 64×64 voxels, of 3.5-mm thickness (no gaps between slices). After completion of the experiment, a T1-weighted anatomical scan was obtained for each subject. Statistical analysis was performed with SPM5 (Statistical Parametric Mapping, http://www.fil.ion.ucl.ac.uk/spm; Wellcome Trust Centre for Neuroimaging, London, UK). The EPI images were realigned, normalized to the Montreal Neurological Institute (MNI) template, smoothed with a 8-mm Gaussian kernel, and filtered temporally with a high-pass cutoff (128 seconds) to remove drift terms. The images were also realigned in time using sinc interpolation before spatial normalization. The data were analyzed in a conventional fashion, using the summary-statistic approach to implement a random effects analysis at the between-subject or second level. At the within-subject level, we specified regressors based on stimulus functions convolved with a hemodynamic response function. These stimulus functions encoded the presentation of each of the three categories of stimuli (events, objects, and persons) and their desirability (desirable, indifferent, and undesirable) based on the subject-specific ratings. Contrasts of category and desirability effects were taken to second-level *t*-tests to produce statistical maps at the group level. Unless otherwise stated, activations were significant at *p*<0.05 corrected family-wise for multiple comparisons at the whole brain level. We also report other activations, where we had an *a priori* reason to expect activity in a specific area; the latter are thresholded at *p*<0.05 corrected within a spherical small volume of 15 mm radius, centred on the cited co-ordinates and are indicated by ‘SVC’ appended to the co-ordinates. For display purposes all figures are thresholded at p<0.001 uncorrected. Co-ordinates are given in MNI space. We also report the cluster size (k_E_) associated with each activation at this display threshold.

Conjunction analyses (CAs) [8] were used to characterize activations common to all three categories. Specifically, we assessed the minimum *t*-statistic from selected contrasts using a conjunction null [9].

### Behavioral data obtained in the scanner (see Table 1)

Table 1A shows the distribution of the classification of stimuli by category averaged across all scanning subjects. A 3×3 chi-square test shows no significant differences, either across response classifications or across stimulus categories (df = 4, χ^2^ = 2.08). A chi-square test was also used to check that subjects made equal numbers of ‘desirable’, ‘indifferent’ and ‘undesirable’ responses in each category and no significant differences were found (events χ^2^ = 3.18, persons χ^2^ = 0.91, objects χ^2^ = 1.46, df in all cases = 2). Table 1B similarly shows response times in classifying a stimulus according to its desirability. A 3×3 (stimulus categories x response classifications) repeated measures ANOVA showed no significant main effect either in stimulus category (F_2,34_ = 0.865) or in response classification (F_2,68_ = 1.195), and also showed no significant interaction between the two factors (F_4,68_ = 0.275). We can conclude that, averaged over all subjects, there were no significant differences in the behavioral data, either between stimulus categories or between response classifications.

**Table 1:** Behavioral data collected in the fMRI study.

(A)	Response classification		
Stimulus category	Undesirable	Indifferent	Desirable
Events	28.24%	30.09%	41.67%
range	(14–30)	(6–33)	(18–42)
Persons	31.56%	37.81%	30.63%
range	(8–45)	(8–50)	(11–43)
Objects	28.78%	32.64%	38.58%
range	(6–41)	(3–46)	(10–50)

(B)	Response classification		
Stimulus category	Undesirable	Indifferent	Desirable
Events	1547.94	1582.78	1578.56
S.D.	299.79	220.69	201.29
range	(1102–2231)	(1124–1942)	(1140–2002)
Persons	1578.94	1590.51	1572.56
S.D.	193.59	149.61	173.84
range	(1246–1874)	(1252–1850)	(1132–1904)
Objects	1513.61	1590.78	1558.28
S.D.	168.16	178.86	200.72
range	(1245–1953)	(1324–1943)	(1043–1854)

(A) Distribution of desirability classifications by stimulus category, averaged over all subjects. Range shows minimum and maximum percentages among subjects. (B) Averaged response times in milliseconds by stimulus category and response classification. S.D. shows sample standard deviation. Range shows minimum and maximum response times, which were averaged within subject, among subjects. Each stimulus category includes 72 trials (a total of 216 trials for a subject).

## Results

### Functional specialization

We first identified activity specific to each stimulus category (events, persons or objects) using statistical parametric maps (SPMs) of the main effect of category. We identified voxels that were significant and specific in showing an increase in activity for one category of pictures over the others by using a conjunction analysis and testing for greater activation in one category relative to the two others.

**Figure 2:**
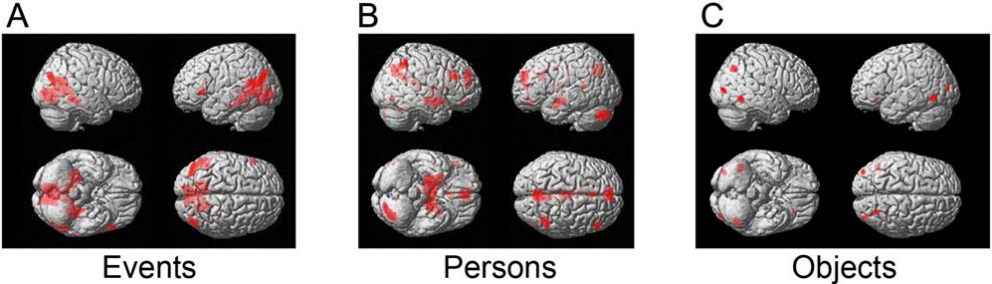
Statistical parametric maps rendered onto a template structural brain, showing category-specific activity as assessed by conjunction analysis, in the comparisons of (A) *events > non-events*, (B) *persons > non-persons*, and (C) *objects > non-objects*.

The distribution of activity in the cortex reflected the functional specialization of the visual brain [10]–[12], since each category produced a distinct pattern of cortical activation (Fig. 2). More specifically:

(Fig. 2A) The CA of *events > non-events* ([*events > persons*] and [*events > objects*]) produced activity bilaterally in the para-hippocampal place area (PPA, [13]) within Brodmann Area (BA) 36, at −28, −42, −8 (*k_E_* = 84) and at 30, −40, −8 (*k_E_* = 24), bilaterally in the angular gyri within BA39 at −36, −76, 30 (*k_E_* = 62) and at 46, −72, 22 (*k_E_* = 32), and in left inferior frontal gyrus (BA44) at −52, 22, 2 (*k_E_* = 12). It also produced responses in the ventral posterior cingulate (BA30) at −12, −56, 12 (*k_E_* = 1514), possibly reflecting the engagement of semantic and episodic memory retrieval systems (e.g., [14]).

(Fig. 2B) The CA of *persons > non-persons* ([*persons > events*] and [*persons > objects*]) produced activity bilaterally in the posterior amygdala at −20, −8, −14 (*k_E_* = 55) and 22, −8, −14 (*k_E_* = 94), which has been implicated in facial perception [15], [16], in the superior frontal gyrus (BA9) at 4, 54, 28 (*k_E_* = 135), in the posterior cingulate (BA23) at 6, −50, 22 (*k_E_* = 22), in the orbito-frontal cortex (**OFC**, BA11) at 2, 40, −16 (*k_E_* = 5), in the left cerebellum at −34, −78, −36 (*k_E_* = 5), and in the thalamus at −2, −10, 8 (*k_E_* = 4). It also produced relatively weak activation in the middle of the fusiform gyrus (BA37) at 44, −56, −24 (*k_E_* = 12) (SVC) corresponding to the fusiform face area (FFA), which has been implicated in the recognition of faces [17], [18]. The relative weakness of the latter activation probably reflects a confound in the stimulus; many of the ‘events’ themselves included people, such as ‘watching TV’ or ‘jogging around a park’ (see also a sample shown Fig 1A), which may have diminished activation in the fusiform face area.

(Fig. 2C) The CA of *objects > non-object*s ([*objects > events*] and [*objects > persons*]) showed activation bilaterally in the occipito-temporal sulcus (BA37) at −44, −64, −6 (*k_E_* = 72) and at 46, −56, −10 (*k_E_* = 80) (both SVC), bilaterally in the lateral occipital gyri, especially centered on V3 (BA19) at −36, −88, 10 (*k_E_* = 27) and 36, −84, 6 (*k_E_* = 43) (both SVC) within the lateral occipital complex [19], and in the right medial parietal lobe (BA7) at 28, −66, 38 (*k_E_* = 55) (SVC). The areas BA37 [20], [21] and V3 (BA19) [22] have been implicated in object and shape recognition, and BA7 in categorizing or discriminating a visual shape from others [23].

### Desirability

**Figure 3:**
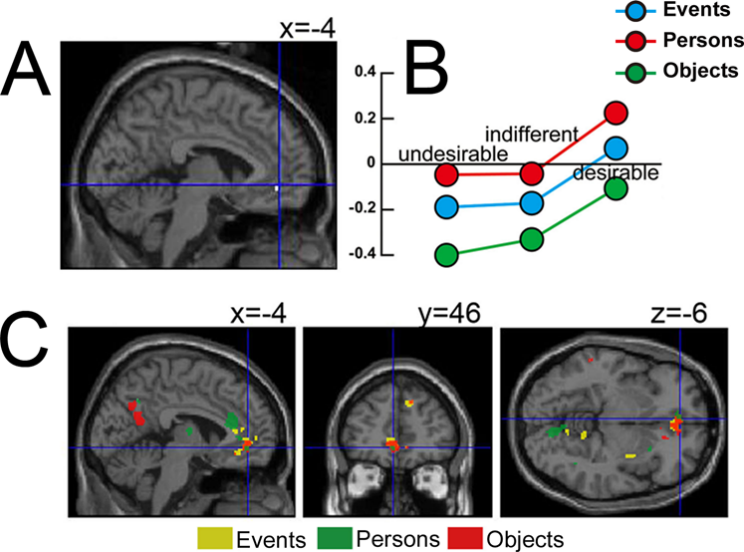
Conjunction analysis of *desirable > undesirable* (displayed at the threshold *P_uncorrected_ <*0.001). Peak activity was located in superior orbito-frontal cortex (−4, 46, −6 SVC) and is shown in sagittal section (A). Parameter estimates for the three stimulus categories and for the three desirability classifications (B). A proximal voxel (−6, 46, −4, see Fig 6A) exhibits a linear relationship with desirability. Figure (C) shows the overlap in areas of cortex activated by each stimulus category (in red, green, and yellow) on sections through a template brain at −4, 46, −6.

The main aim of the present study was to investigate the neural correlates of desire in general. The CA of *desirable > undesirable*, in which three contrasts (*desirable > undesirable* for events, persons, and objects) were analyzed to extract common activations, disclosed the superior orbito-frontal cortex (**SOFC**; BA11) (−4, 46, −6; *k_E_* = 14, SVC) (Fig. 3A). Fig. 3C shows that there is an overlap in the activity produced by the CA of *desirable > undesirable* for each stimulus category.

**Figure 4:**
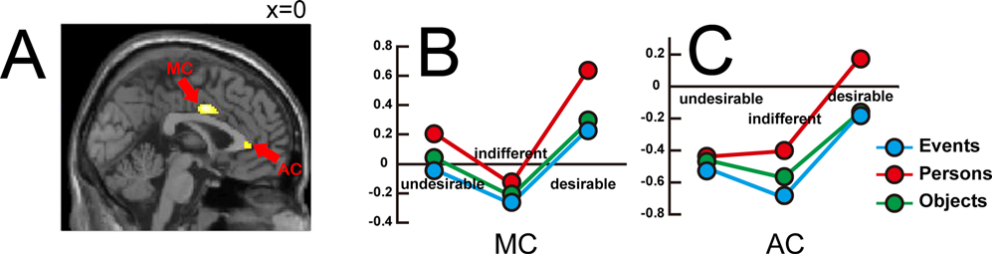
Conjunction analysis of *desirable > indifferent* (displayed at the threshold *P_uncorrected_ <*0.001). Peak activity was located in the mid-cingulate cortex (MC above) (0, 2, 36, SVC). Another activation was in anterior cingulate cortex (AC above) (0, 34, 4 SVC). Both are shown in sagittal section (A). Parameter estimates for the three stimulus categories and for the three desirability classifications for MC (B) and for AC (C). The peak activity voxel in MC at 0, 2, 36 falls within the cluster of active voxels shown in Fig 6B which exhibit a quadratic relationship with desirability.

The CA of *desirable > indifferent* activated two distinct zones, one in the mid-cingulate gyrus (**MC**) (BA24) at 0, 2, 36, SVC (*k_E_* = 85) and the other in the anterior cingulate (**AC**) cortex (BA32) at 0, 34, 4, SVC (*k_E_* = 18) (Fig. 4A).

The CA of contrasts testing *indifferent > desirable*, *indifferent > undesirable*, *undesirable > desirable*, and *undesirable > indifferent*, revealed no significant conjunctions.

The only activations common to all stimulus categories were in superior orbito-frontal and cingulate cortices (middle and anterior). In conclusion, it would seem that activity in these cortical areas correlates with classifying a stimulus according to its desirability.

### Response modulations

The SOFC at −4, 46, −6 (Fig. 3A) (active in the CA of *desirable > undesirable*), the MC at 0, 2, 36 and the AC at 0, 34, 4 (Fig. 4A) (both active in the CA of *desirable > indifferent*), showed BOLD signal changes for all three stimulus categories and three response classifications (see Fig. 3B, Fig. 4B and 4C respectively).

In the SOFC (Fig. 3B) the weakest activation was for stimuli classified as ‘undesirable’ and the strongest for those classified as ‘desirable’, with activity for those classified as ‘indifferent’ lying in between. In the MC (Fig. 4B) there was much greater activity for both ‘desirable’ and ‘undesirable’ than for ‘indifferent’ responses, irrespective of stimulus categories. In the AC (Fig. 4C) activity to ‘desirable’ was greater than activity to ‘indifferent’.

**Figure 5:**
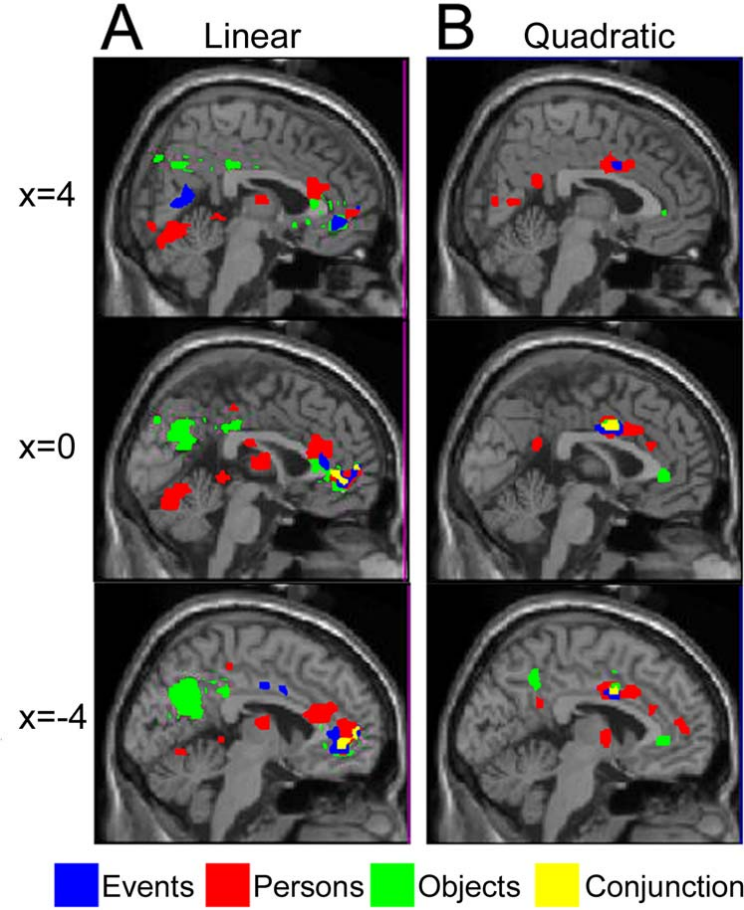
Areas in which brain activity for three stimulus categories showed parametric modulation by desirability rating (*P_uncorrected_ <*0.001); (A) linear (1^st^ order) relationship, (B) quadratic (2^nd^ order) relationship. Activations are shown superimposed on successive sagittal sections (x = −4, 0, 4).

**Figure 6:**
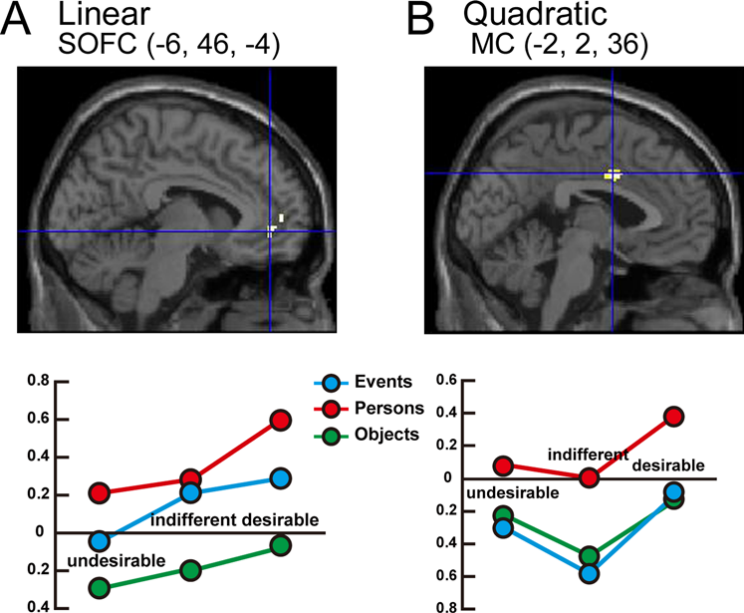
Locations shown in sagittal section and parameter estimates showing modulation by desirability rating (A) linear (1^st^ order) relationship in SOFC at −6, 46, −4, (B) quadratic (2^nd^ order) relationship in MC at −2, 2, 36.

To investigate the distribution of these relationships, we carried out a separate analysis in which we modeled the regressors for the three categories (events, persons and objects) with desirability as a parametric modulator. Desirability was coded as 0, 1 and 2 for ‘undesirable’, ‘indifferent’ and ‘desirable’ respectively, and 1^st^ and 2^nd^ order polynomial expansion elements were included in the parametric modulation. We carried out a similar conjunction analysis across the three categories (Fig. 5) and found that only the SOFC (peak −6, 46, −4; *k_E_* = 56, SVC) exhibits a linear relationship (Fig. 6A) while the MC (peak −2, 2, 36; *k_E_* = 44, SVC) exhibits a quadratic dependency on desirability (Fig. 6B). These locations differ very slightly from the locations discovered in the CAs of *desirable > undesirable* (−4, 46 −6; Fig. 3) and *desirable > indifferent* (0, 2, 36; Fig. 4) but nevertheless lie within the corresponding cluster groups. When the desirability rating was coded in the opposite sense (2, 1 and 0 for ‘undesirable’, ‘indifferent’ and ‘desirable’ respectively) we found no areas exhibiting either a 1^st^ order or a 2^nd^ order relationship.

## Discussion

In our experiments, we asked subjects to classify objects, events, or persons as to their desirability, not their preference. A subject might categorize a stimulus as undesirable when on a full stomach at scan time, even if he or she likes the specific food stimulus (e.g., cake) presented by the visual image; heterosexual men might categorize the image of a male actor as ‘undesirable’ even if they have a preference for the actor and the movie. Disentangling desirability from preference is important but not easy (see [24]). Unfortunately, the distinction is difficult to make neurologically, at least in humans. While recent animal (rat) studies have shown that responses in the nucleus accumbens (NAc) and the ventral pallidum (VP) correlate with hedonic ‘liking’ reactions to sweetness, the NAc dominated appetitive ‘wanting’ reactions independently of VP activation [25]. In humans, Rolls and McCabe [26] have shown high correlations with pleasantness ratings in chocolate cravers and non-cravers in medial OFC activity, with the non-craver group showing smaller effects. By contrast, they did not find such correlations with ‘wanting’ ratings in either group. Even so, the distinction is worth keeping in mind for future studies of this fundamental state.

### Functional specialization

In this study, we explored brain areas that are specialized for categorizing a stimulus according to its desirability. We found, as in our previous study of beauty [27], that different categories of stimuli (e.g., persons or events) activated distinct parts of the visual brain, thus emphasizing the generality of the functional specialization of the visual brain, seen even at its earliest stages [10]–[12]. This strongly suggests that these specialized areas are participants in the classification, feeding their inputs into distinct areas of the brain which, subsequently, abstract the quality of desirability, since these latter areas do not show any evident specialization, their responses correlating with desirability instead, regardless of the source. This result is similar to our recent study of brain activity in response to temporally irregular patterns, where the irregular patterns generated activity in different regions of the occipital lobe depending upon whether they were generated by color, luminance, number or letter stimuli; by contrast, there was a common area in the frontal lobe that was active regardless of the category of stimulus that generated the irregular pattern [28].

### Activity in the brain's reward system

In addition, other cortical areas related to the reward system of the brain were also activated; in one (SOFC; Fig. 6A), the activity is related linearly to the declared intensity of the experience of desire while in another (area MC in Fig. 6B) the relationship is quadratic, implying that it registers whether the stimulus is desirable or undesirable without classifying it further. Activity in a third area (area AC in Fig. 4A) correlated with the contrast *desirable > indifferent* without a significant linear or quadratic modulatory effect. The cortex extending between the OFC and the medial cingulate cortex has many subdivisions, which may be involved in different aspects of social, emotive, and cognitive processing [29], [30], [31], [32]. The number of functionally distinct areas within Brodmann areas 11, 12, 24, and 32 and the connections between them and sub-cortical regions such as the ventral tegmental area or the ventral striatum/nucleus accumbens remains unknown. This expanse of cortex is associated with a variety of emotional states related to reward and motivation, such as monetary reward [1]–[3], drug craving (e.g., [4], [5] see also [33], [34] for reviews), sexual arousal (e.g., [35], [36]), food eating or choice (e.g., [6], [7]), beauty [27], and love [37], [38]. Overall, the results of the present study are similar to results obtained in antecedent studies by us [27] and other groups (e.g. [2], [39], [40], [41], [42]) in showing that activity in some of these areas (as in the SOFC in this study) is related linearly to the declared intensity of experience while in others (represented in this study by MC) it is not, ‘desirable’ and ‘undesirable’ stimuli eliciting greater activity than indifferent ones. AC, by contrast, was only active to stimuli classified as ‘desirable’.

### A. The orbito-frontal cortex

The OFC is part of the reward circuitry, where the assessment of facial beauty or attractiveness [39], [43], [44], of pleasurable response to music [40] and of pleasant touch [45] seems to be registered. Other evidence relating to the graded response of this area, in relation to intensity of subjective experience, comes from the subjective evaluation of the attractiveness of faces [46], [47]. BOLD activity was found to relate to the experienced attractiveness and, interestingly, related to gender- and sexual orientation-based evaluation of attractiveness; thus activation of OFC was more pronounced when heterosexual men and homosexual women rated a female face as attractive or when heterosexual women and homosexual men rated male faces as attractive.

It is interesting that the OFC has also been implicated in emotional disorders, including depression (e.g., [48]). Patients with depression commonly exhibit symptoms of loss of interest, energy, motivation, pleasure and/or sexual interest (e.g., [49], [50]). Antidepressants enhance noradrenergic and dopaminergic activity to offset these symptoms (e.g., [51]). Our evidence is thus consistent with other studies in highlighting the OFC as a cortical area whose healthy functioning correlates with states of desire and whose abnormal physiology may lead to a loss of motivation and desire, of which the general loss of the ability to experience pleasure and the specific loss of sexual desire (i.e. libido) are key symptoms.

### B. The cingulate cortex

The other two areas that responded when stimuli were judged to be desirable were located in cingulate gyrus. In one area (area MC in Fig. 4), the desirable and undesirable states gave a higher response than the indifferent judgment. In fact, in a previous study this area was found to be active in response to both pleasant and unpleasant odours compared to clean air, in contrast to the OFC which was activated by pleasant odours only [52]. Similarly, the function of MC in our study would appear to be quite distinct, and only related to the experience, not its intensity. Interestingly, only desirable or undesirable stimuli induce the need for potential response selection (approach or avoid respectively), a role this area has been implicated in [31], [32], [53]. Similar responses can be recorded from the amygdala, which responds to a broad range of emotions relative to stimuli of neutral valence [54], [55]. The amygdala also responds with equal intensity to attractive and unattractive faces, relative to those of medium attractiveness [41]. This suggests that such structures are tuned to the detection of events of emotional value irrespective of valence in the sensory environment [56], [57]. The other area (area AC in Fig. 4) showed a higher response for ‘desirable’ than for ‘indifferent’. This area was also active in our previous beauty study in the comparison beautiful versus neutral, but not ugly versus neutral [27]. Another area engaged in a similar way is the angular gyrus which is associated with spatial attention [58]. This region was active in the comparison beautiful versus neutral, which may have placed a greater load on the attentional system [56] but its responses, unlike the OFC but like AC, were not graded with the declared intensity of the beauty experienced [27].

### Other activations

In addition to the common activation of SOFC, there were activations that were particular to each category of visual stimulus (Fig. 2). Previously, drugs and monetary rewards have been shown to activate regions such as the nucleus accumbens (NAc), the striatum, and the hypothalamus (e.g., [59], [60]). Although these areas were partially activated with each stimulus category in the present study (e.g., object in NAc, persons in hypothalamus) the precise neural network activated is not identical for the different categories, implying that each has a unique neural signature. However, these more specialized activations are not of present interest to us, in work whose aim was to locate the cortical areas that correlate with desirability in general.

### Summary and conclusions

In summary, this study shows that categorizing a stimulus according to its desirability activates specific regions in the reward system associated with general appeal to the subjects, regardless of stimulus categories. We did not, however, detect any area whose activity correlated specifically with stimuli judged to be undesirable. This suggests a push-pull mechanism correlated to desire, in which the two areas in the cingulate gyrus and the orbito-frontal cortex mediate or modulate both positive and negative desirability. We did not observe any activity in the caudate nucleus, putamen, thalamus or nucleus accumbens that might relate inversely with activity in the orbito-frontal cortex. This, too, might indicate that such a reciprocal relationship might be embedded in a push-pull mechanism that dictates the response of the orbito-frontal cortex.

The results have thus brought us a little closer to understanding the neural basis of one of the most important aspects of human conduct, namely goal directed behavior towards attaining pleasurable experiences. Desire may have a somewhat different connotation for those influenced by Oriental culture than for those reared in a Western culture. Those reared in a tradition that commonly - under the influence of Buddhism – regards desire as an unwelcome distraction from achieving a state of happiness might view desire in a different light than those nourished in a Western culture, although of course desire is often also regarded as ‘undesirable’ in the latter culture. All our subjects were Japanese and we did not question them as to their attitudes towards desire. It is possible, though we think unlikely, that the results we describe here are not applicable to others from different cultural traditions. It is a question we will address in future studies.
